# Masquerading Spleen: A Perplexing Case of Intrahepatic Splenosis Mimicking Hepatocellular Carcinoma

**DOI:** 10.7759/cureus.70145

**Published:** 2024-09-25

**Authors:** Noriko Ikeda, Yujo Kawashita, Masaki Tateishi, Takashi Ueda, Junzo Yamaguchi, Yasuo Washida, Yoichi Hachitanda

**Affiliations:** 1 Surgery, Fukuoka Seishukai Hospital, Fukuoka, JPN; 2 Radiology, Fukuoka Seishukai Hospital, Fukuoka, JPN; 3 Pathology, Fukuoka Seishukai Hospital, Fukuoka, JPN

**Keywords:** hepatectomy, hepatocellular carcinoma, intrahepatic splenosis, liver tumor, splenic trauma

## Abstract

Intrahepatic splenosis is an uncommon condition that can present a significant diagnostic challenge, often masquerading as more sinister hepatic lesions. We report a perplexing case of a 56-year-old female with a history of splenectomy who presented with liver masses initially suspected to be hepatocellular carcinoma (HCC). Despite advanced imaging techniques, including ultrasonography, computed tomography (CT), and magnetic resonance imaging (MRI), the lesions convincingly mimicked HCC. Surgical resection was performed, and histopathological examination revealed the true nature of the masses: intrahepatic splenosis. This case underscores the importance of considering this rare entity in the differential diagnosis of liver masses, particularly in patients with a history of splenic trauma or splenectomy. We present a review of the literature to provide context and discuss the diagnostic conundrum posed by intrahepatic splenosis.

## Introduction

Splenosis, the autotransplantation of splenic tissue following trauma or surgery, is a well-documented phenomenon that continues to intrigue clinicians and researchers alike [1]. While commonly occurring in the abdominal cavity, intrahepatic splenosis represents an exceptionally rare manifestation of this condition, with fewer than 100 cases reported in the medical literature to date [2].

The true incidence of intrahepatic splenosis remains elusive due to its rarity and often asymptomatic nature [3]. The diagnostic challenge posed by intrahepatic splenosis lies in its remarkable ability to mimic primary or metastatic liver tumors in imaging studies [4]. This uncanny resemblance can lead to a cascade of unnecessary invasive procedures or surgical interventions [5].

We present a perplexing case of intrahepatic splenosis that masterfully impersonated hepatocellular carcinoma (HCC), highlighting the critical importance of maintaining a high index of suspicion for this condition in patients with a history of splenic trauma or splenectomy.

## Case presentation

A 56-year-old female patient with a history of splenectomy presented to our hospital for evaluation of newly discovered liver masses. The patient’s history was significant for heavy alcohol consumption, averaging approximately 1400 mL of beer daily. Her past medical history included postoperative status for ventricular septal defect repair, cholecystectomy, and cesarean section. Notably, she had undergone a splenectomy in her 30s due to a splenic abscess that developed as a complication following her cesarean section.

Laboratory investigations revealed mildly elevated liver enzymes with aspartate aminotransferase (AST) 40 U/L and alanine aminotransferase (ALT) 34 U/L. Tumor markers were within normal ranges, with alpha-fetoprotein (AFP) at 11.6 ng/mL and protein induced by vitamin K absence or antagonist-II (PIVKA-II) at 35 AU/mL. Hepatitis B and C viral markers were negative.

**Figure 1:**
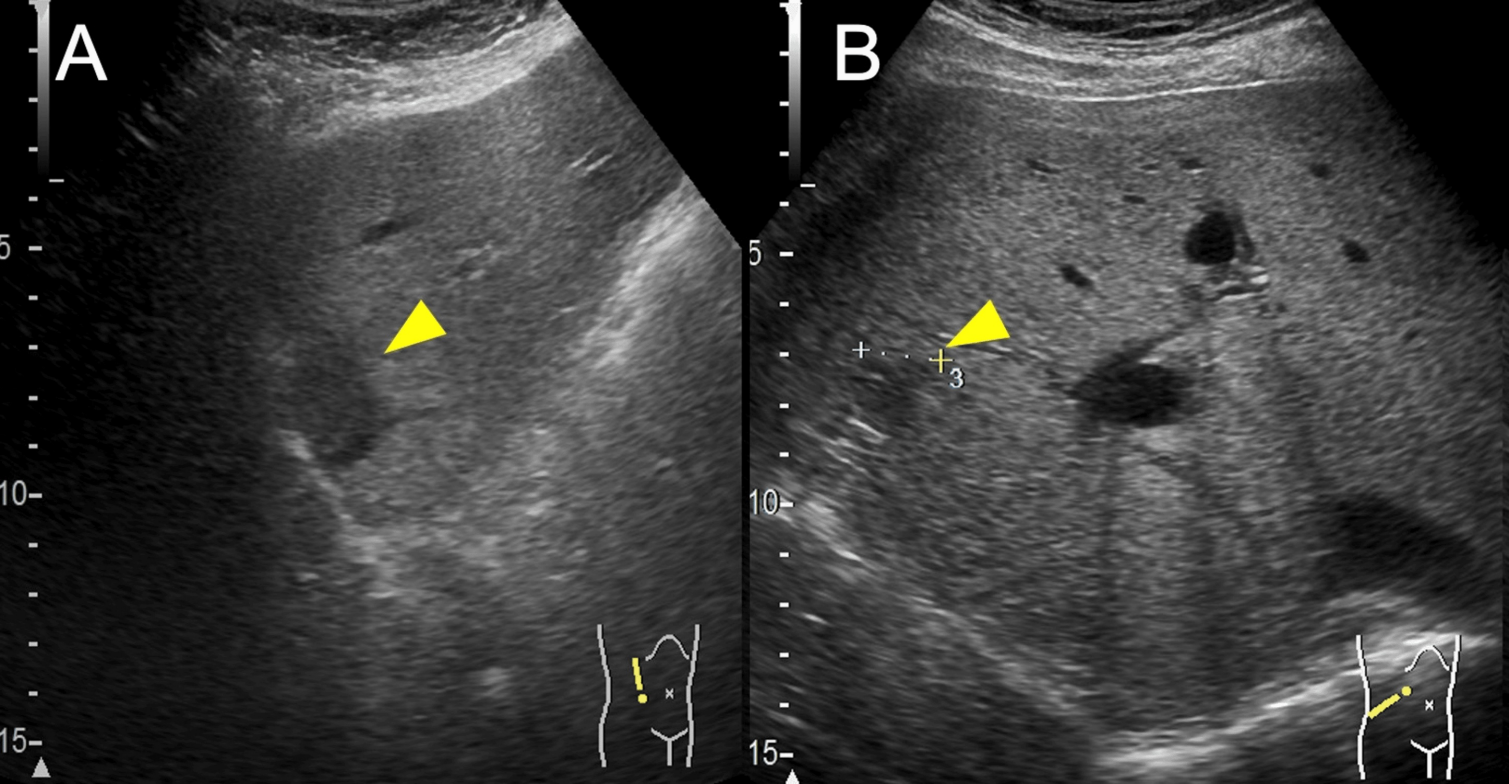
Ultrasonography image of the liver (A) Ultrasonography image demonstrating a hypoechoic mass in segment 2 of the liver (yellow arrowhead). (B) Ultrasonography image showing a hypoechoic mass in segment 6 of the liver (yellow arrowhead). Both lesions appear well-demarcated with homogeneous internal echoes, measuring approximately 2 cm in diameter. The surrounding liver parenchyma shows a coarse echotexture, consistent with chronic liver disease.

Abdominal ultrasonography revealed hypoechoic masses in segments 2 and 6 of the liver. Both masses measured 2 cm in diameter (Figure 1).

**Figure 2:**
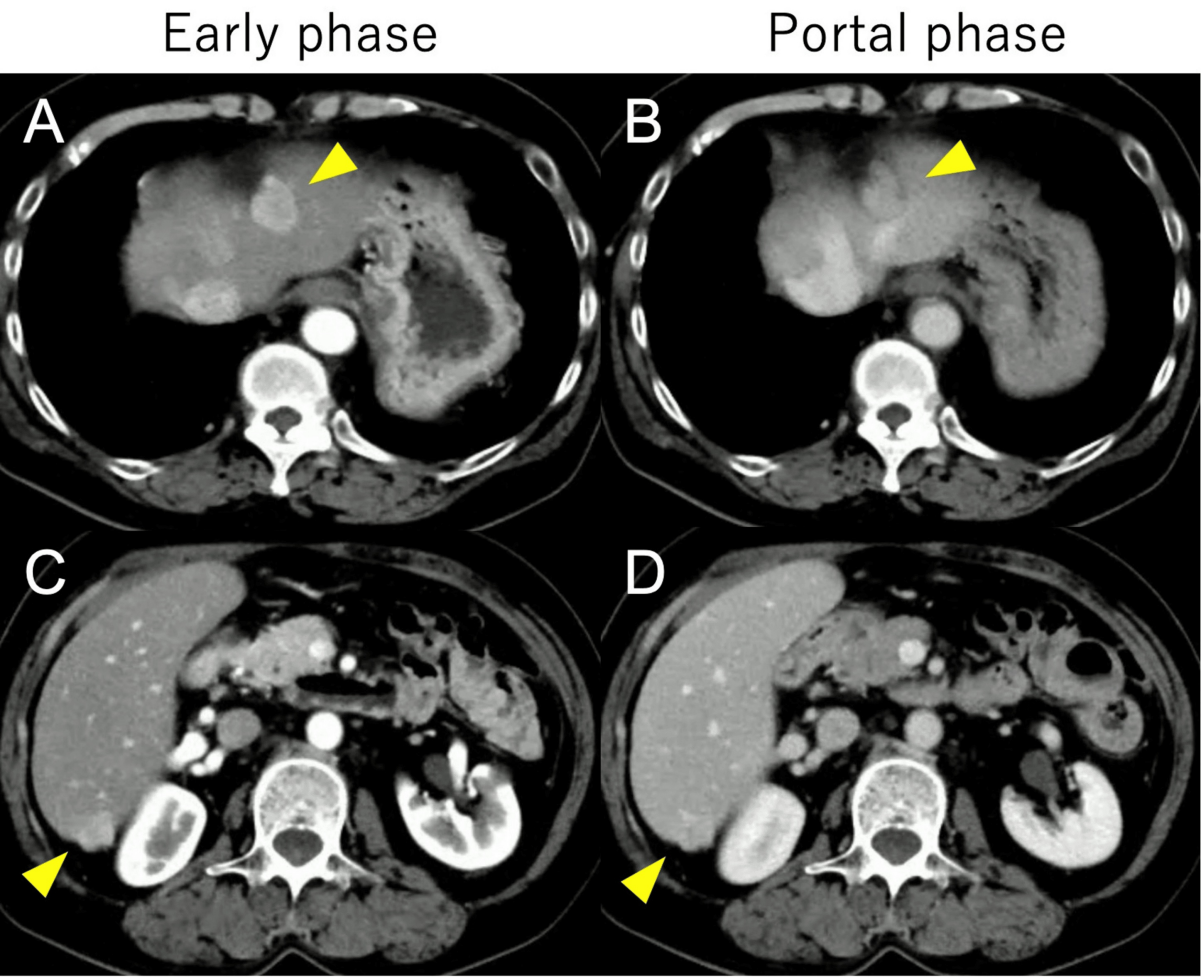
Dynamic CT images of the liver (A, B) Early arterial phase (A) and portal phase (B) of the upper abdomen, show a homogeneously enhancing mass in segment 2 of the liver (yellow arrowheads). The lesion demonstrates higher attenuation compared to the surrounding liver parenchyma in the early phase (A) and becomes relatively hypoattenuating in the portal phase (B). (C, D) Early arterial phase (C) and portal phase (D) of the lower abdomen, reveal a similar enhancing mass in segment 6 of the liver (yellow arrowheads). This lesion also shows hyperattenuation in the early phase (C) and relative hypoattenuation in the portal phase (D). Both lesions demonstrate enhancement patterns mimicking the washout typically seen in hepatocellular carcinoma, with hyperenhancement in the early arterial phase and relative hypoattenuation in the portal venous phase compared to the surrounding liver parenchyma.

Dynamic CT demonstrated homogeneous enhancement of the masses in the arterial phase, followed by washout in the portal and delayed phases (Figure 2).

**Figure 3:**
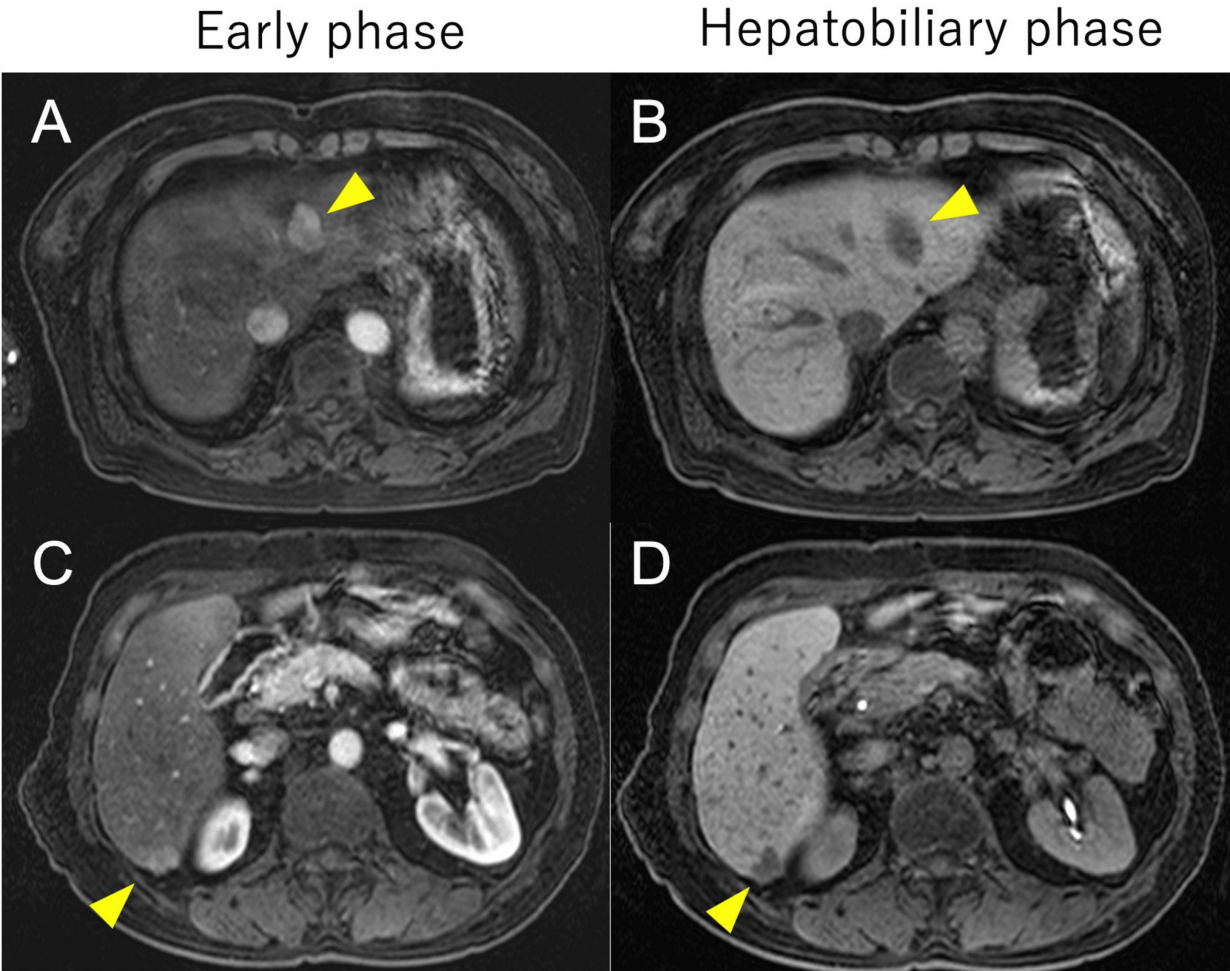
Gadoxetic acid-enhanced MRI (EOB-MRI) of the liver (A, C) Early arterial phase images showing hyperintense enhancement of the lesions in segment 2 (A, yellow arrowhead) and segment 6 (C, yellow arrowhead) of the liver. (B, D) Hepatobiliary phase (20 minutes post-contrast) demonstrating hypointensity of the lesions in segment 2 (B, yellow arrowhead) and segment 6 (D, yellow arrowhead) against the background of enhancing liver parenchyma. Both lesions demonstrate enhancement patterns often associated with hepatocellular carcinoma, showing hyperintensity in the early arterial phase and hypointensity in the hepatobiliary phase compared to the surrounding liver parenchyma.

Gadoxetic acid-enhanced MRI (EOB-MRI) showed early arterial enhancement of the lesions with hypointensity in the hepatobiliary phase, a pattern often associated with HCC (Figure 3).

Given the patient’s history of alcohol abuse and the compelling imaging findings, HCC could not be confidently excluded. Consequently, surgical resection of liver segments 2 and 6 was performed.

**Figure 4:**
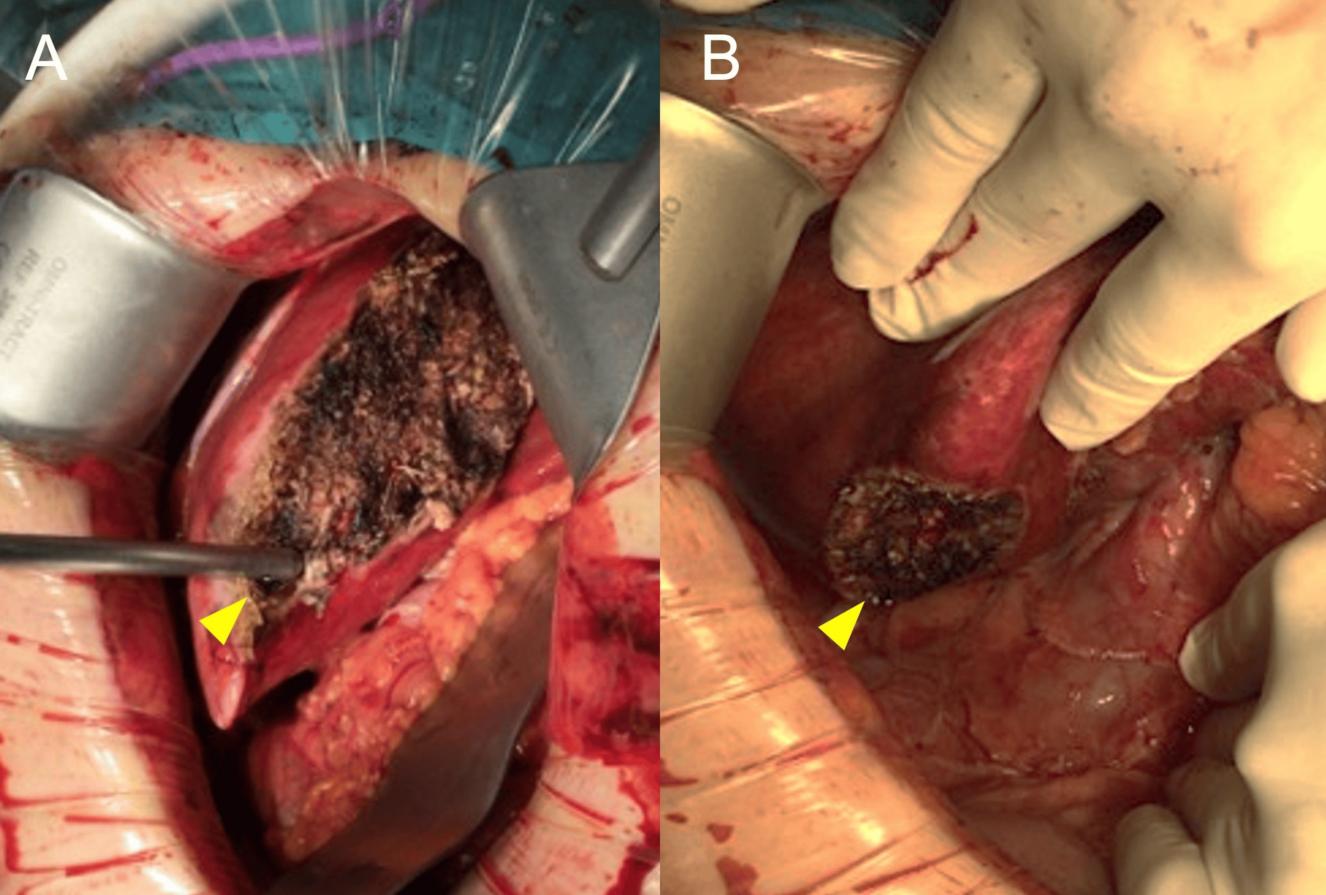
Intraoperative images of liver resection (A) Resection surface after partial hepatectomy of segment 2. The yellow arrowhead indicates the cut surface where the tumor was removed, demonstrating its superficial location. (B) Resection surface following partial hepatectomy of segment 6. The yellow arrowhead points to the area where the tumor was excised, also showing its proximity to the liver surface. Both images illustrate that the tumors were located relatively close to the liver surface, which facilitated successful partial hepatectomy. The exposed liver parenchyma and the surgical margins are clearly visible in both resection beds.

Intraoperative findings revealed well-demarcated, dark-red lesions in the resected liver segments (Figure 4).

**Figure 5:**
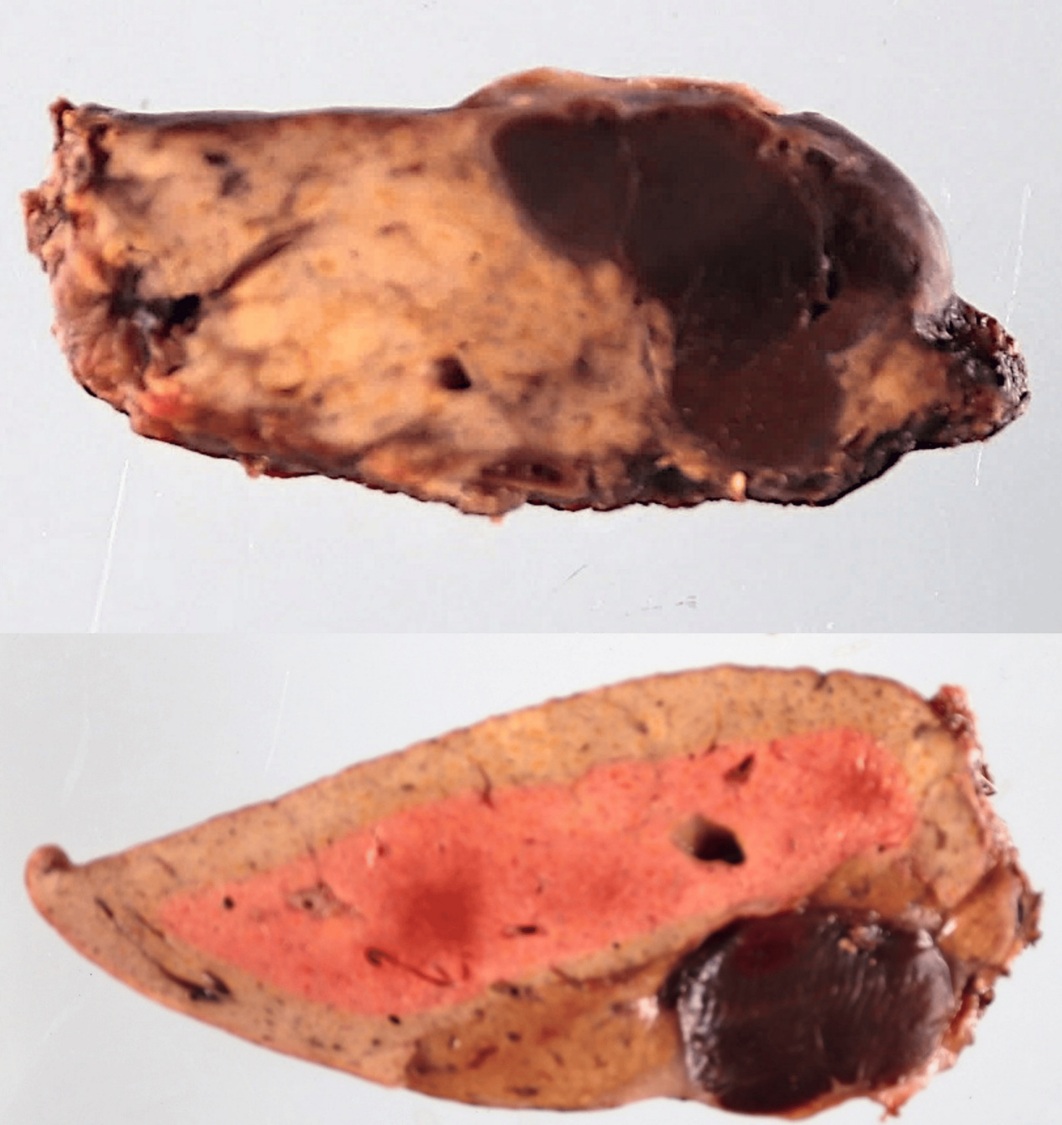
Gross pathological specimen of the resected liver segment Gross pathological specimens of the resected liver segments show the lesions. The cut surfaces reveal two well-circumscribed, dark-red nodules, each measuring approximately 2 cm in diameter. The nodules' color and texture are consistent with splenic tissue, contrasting sharply with the surrounding liver parenchyma.

A gross pathological examination showed two well-circumscribed, dark-red nodules, each measuring approximately 2 cm in diameter (Figure 5).

**Figure 6:**
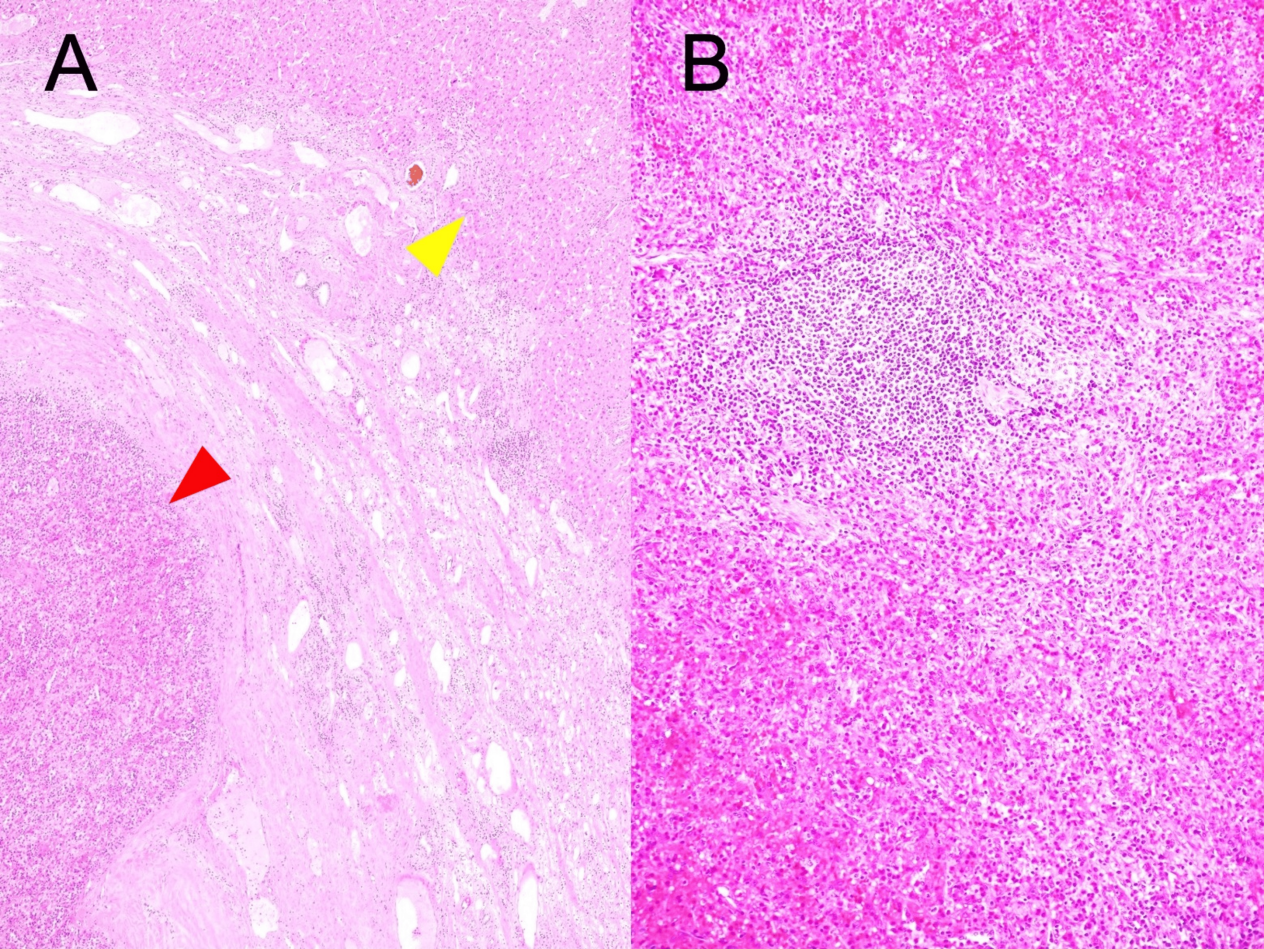
Histopathological images confirming intrahepatic splenosis (A) Low-power view (Hematoxylin and eosin (H&E) stain) showing the interface between liver tissue (yellow arrowhead) and splenic tissue (red arrowhead). The clear demarcation between hepatic and splenic architecture is evident. (B) Higher magnification view (H&E stain) of the splenic tissue, demonstrating characteristic splenic architecture including white pulp (lymphoid follicles) and surrounding red pulp. These histopathological findings confirm the diagnosis of intrahepatic splenosis.

Histopathological examination of the resected specimens showed splenic tissue within the liver parenchyma, confirming the unexpected diagnosis of intrahepatic splenosis (Figure 6).

The patient’s postoperative course was uneventful, with no complications observed. She recovered smoothly and was discharged from the hospital in good condition on the seventh postoperative day.

## Discussion

Intrahepatic splenosis presents a fascinating diagnostic enigma, skillfully masquerading as HCC in imaging studies [6]. This case exemplifies the challenges inherent in differentiating this benign condition from malignant hepatic lesions, particularly in the context of a patient with risk factors for HCC. Similar diagnostic dilemmas have been reported by Oyama et al. [7] and Di Cataldo et al. [8], where intrahepatic splenosis was initially misdiagnosed as HCC in patients with a history of splenectomy. These cases underscore the difficulty in differentiating intrahepatic splenosis from HCC based on imaging alone. However, our patient's history of chronic alcohol abuse adds a unique aspect, potentially contributing to the development and growth of the ectopic splenic tissue. These experiences collectively emphasize the importance of considering a patient's full medical history, including any history of splenic trauma or splenectomy, when evaluating liver lesions.

It is important to note that in cases of suspected HCC with potentially resectable liver tumors, we typically do not perform needle biopsies due to the risk of tumor implantation along the needle track. This precautionary approach, while reducing the risk of tumor seeding, can sometimes lead to diagnostic challenges as seen in our case.

The development of intrahepatic splenosis involves complex mechanisms that are not yet fully elucidated. Two principal theories have been proposed: direct seeding and hematogenous spread [9]. Understanding these mechanisms is crucial for recognizing the potential locations and appearances of this elusive condition.

**Figure 7:**
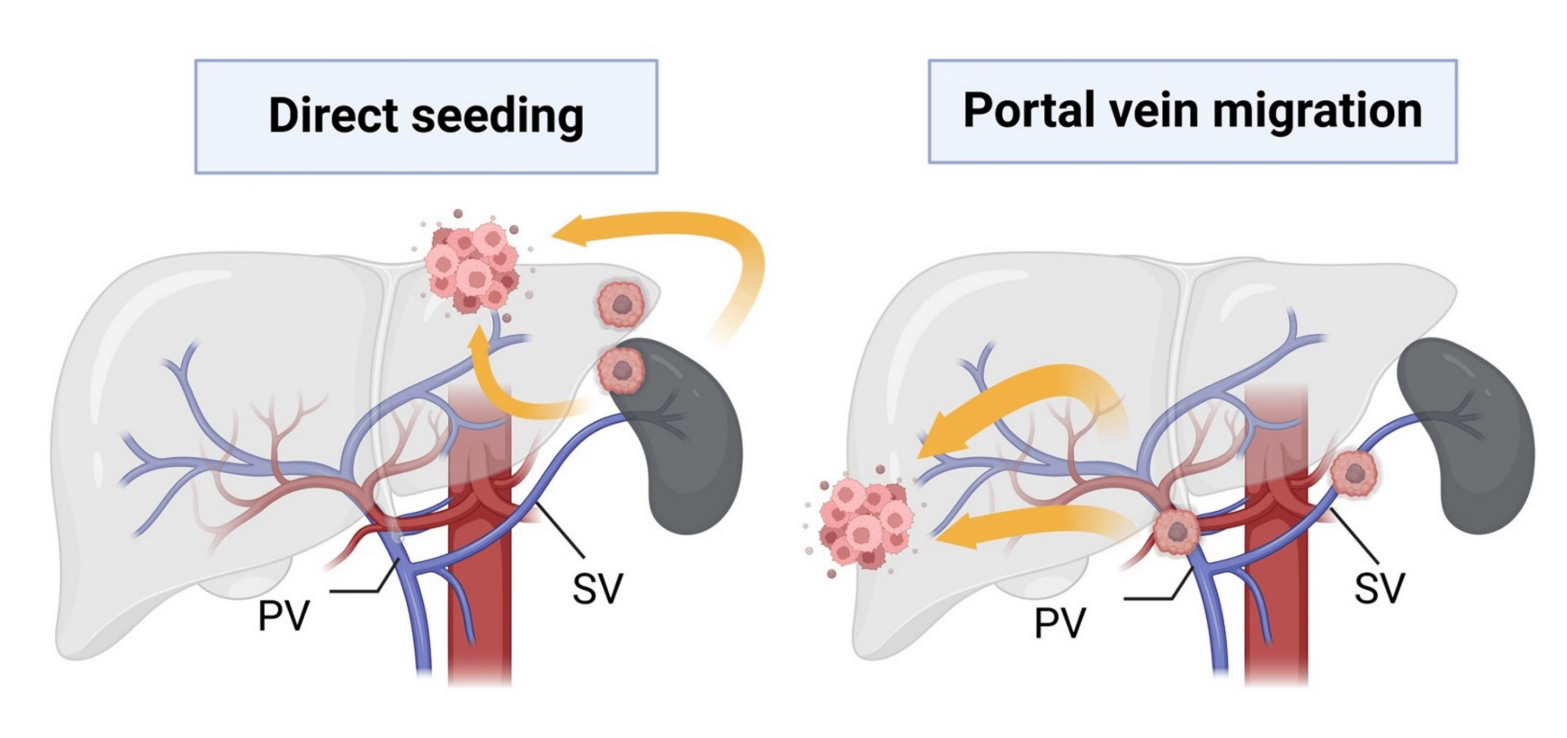
Proposed model for the development of intrahepatic splenosis PV: portal vein; SV: splenic vein Diagram illustrating the proposed model for the development of intrahepatic splenosis in the context of chronic alcohol abuse. The model shows the progression from normal liver to alcohol-induced chronic inflammation, creating a permissive microenvironment for splenic cell engraftment. Subsequent stages depict the implantation, survival, and growth of ectopic splenic tissue within the inflamed liver parenchyma, facilitated by the altered local environment. Image credit: Created in BioRender. Noriko, I. (2024) BioRender.com/n19e308

In our case, we propose a novel theory that may explain the development and growth of intrahepatic splenosis in this particular patient. The patient's history of chronic alcohol abuse likely resulted in ongoing liver damage and inflammation. We hypothesize that this chronic hepatic insult may have created a microenvironment conducive to the engraftment and proliferation of splenic cells. The constant stimulation from alcohol-induced liver injury could have promoted the survival and expansion of ectopic splenic tissue within the liver parenchyma (Figure 7).

This theory is supported by recent research on liver fibrosis and repair mechanisms. Pellicoro et al. have described the complex immune regulation of wound healing in the liver, highlighting how chronic inflammation can alter the hepatic microenvironment [10]. These alterations could potentially create niches favorable for the engraftment and survival of ectopic splenic tissue. Furthermore, Forbes and Rosenthal have discussed how tissue damage can prepare the ground for regeneration, a process that might be exploited by splenic cells in the context of intrahepatic splenosis [11].

The imaging characteristics of intrahepatic splenosis can be remarkably similar to those of HCC, creating a diagnostic quagmire that often leads to unnecessary interventions. On ultrasound, lesions typically appear hypoechoic, a finding that is nonspecific and can be seen in various focal liver lesions [12]. CT imaging often shows homogeneous enhancement in the early phase, persisting into the portal phase, a pattern that differs subtly from the classic washout seen in HCC [13]. This persistent enhancement is thought to reflect the unique vascular architecture of splenic tissue, which includes sinusoidal spaces that allow for prolonged contrast retention.

MRI findings in intrahepatic splenosis can be variable, but lesions often show hypointensity on T1-weighted images and hyperintensity on T2-weighted images [14]. The use of hepatocyte-specific contrast agents, such as gadoxetic acid (Gd-EOB-DTPA), has added another layer of complexity to the differentiation between intrahepatic splenosis and HCC. In our case, the EOB-MRI findings of early arterial enhancement and hypointensity in the hepatobiliary phase were convincingly suggestive of HCC, leading to the decision for surgical resection. This highlights the limitations of even advanced imaging techniques in definitively characterizing these lesions.

To avoid unnecessary surgical interventions, it is crucial to maintain a high index of suspicion for intrahepatic splenosis when evaluating liver masses, especially in patients with a history of splenic trauma or splenectomy [15]. Additional diagnostic tools can be invaluable in confirming the diagnosis of this elusive condition. Tc-99m heat-damaged red blood cell scintigraphy, for instance, has shown high sensitivity and specificity for detecting ectopic splenic tissue [16]. This nuclear medicine technique takes advantage of the unique ability of splenic tissue to take up damaged red blood cells, providing a functional assessment that can complement anatomical imaging.

The management of intrahepatic splenosis remains a subject of debate. While the condition is generally considered benign, there have been rare reports of complications such as infarction or rupture of ectopic splenic tissue [17]. Additionally, the potential immunological benefits of functional splenic tissue in post-splenectomy patients must be weighed against the risk of misdiagnosis and unnecessary intervention. In asymptomatic patients with confirmed intrahepatic splenosis, conservative management with regular imaging follow-up is often recommended [4].

## Conclusions

This case underscores the diagnostic challenge of intrahepatic splenosis mimicking HCC, particularly in patients with a history of splenic trauma or splenectomy. It highlights the limitations of current imaging modalities and emphasizes the need for increased awareness among clinicians. Utilizing specialized diagnostic techniques may reduce unnecessary interventions. Further research into the management of intrahepatic splenosis is essential to improve clinical decision-making in such diagnostically challenging cases.
